# How to build a fruit: Transcriptomics of a novel fruit type in the Brassiceae

**DOI:** 10.1371/journal.pone.0209535

**Published:** 2019-07-18

**Authors:** Shane Carey, Kerrin Mendler, Jocelyn C. Hall

**Affiliations:** 1 Biological Sciences, University of Alberta, Edmonton, AB, Canada; 2 Department of Biology, University of Waterloo, Waterloo, Ontario, Canada; Youngstown State University, UNITED STATES

## Abstract

Comparative gene expression studies are invaluable for predicting how existing genetic pathways may be modified or redeployed to produce novel and variable phenotypes. Fruits are ecologically important organs because of their impact on plant fitness and seed dispersal, modifications in which results in morphological variation across species. A novel fruit type in the Brassicaceae known as heteroarthrocarpy enables distinct dispersal methods in a single fruit through segmentation via a lateral joint and variable dehiscence at maturity. Given the close relationship to Arabidopsis, species that exhibit heteroarthrocarpy are powerful models to elucidate how differences in gene expression of a fruit patterning pathway may result in novel fruit types. Transcriptomes of distal, joint, and proximal regions from *Erucaria erucarioides* and *Cakile lanceolata* were analyzed to elucidate within fruit and between species differences in whole transcriptome, gene ontology, and fruit patterning expression profiles. Whole transcriptome expression profiles vary between fruit regions in patterns that are consistent with fruit anatomy. These transcriptomic variances do not correlate with changes in gene ontology, as they remain generally stable within and between both species. Upstream regulators in the fruit patterning pathway, *FILAMENTOUS FLOWER* and *YABBY3*, are expressed in the distal and proximal regions of *E*. *erucarioides*, but not in the joint, implicating alterations in the pathway in heteroarthrocarpic fruits. Downstream gene, *INDEHISCENT*, is significantly upregulated in the abscissing joint region of *C*. *lanceolata*, which suggests repurposing of valve margin genes for novel joint disarticulation in an otherwise indehiscent fruit. In summary, these data are consistent with modifications in fruit patterning genes producing heteroarthrocarpic fruits through different components of the pathway relative to other indehiscent, non-heteroarthrocarpic, species within the family. Our understanding of fruit development in Arabidopsis is now extended to atypical siliques within the Brassicaceae, facilitating future studies on seed shattering in important Brassicaceous crops and pernicious weeds.

## Introduction

Studying gene expression patterns across plant structures and species can elucidate how their modification may produce morphological variation [1,2]. Fruits are diverse and ecologically relevant plant structures to investigate because their morphological variation determines how their seeds are dispersed [3,4]. There are multitudinous fruit morphologies in nature, and they are often categorized as fleshy or dry. Fleshy fruits are distributed primarily by animals, as the seeds are discarded before or after consuming. Dry fruits however, may be dispersed by animals, wind, or water. Dry fruits are further classified by whether they are dehiscent, releasing seeds into the environment, or indehiscent, releasing seeds in a protected fruit wall propagule. Thus, variation in fruit morphology is directly tied to differences in dispersal capabilities.

Brassicaceae is an exemplary group to investigate the basis of fruit diversity because species in this family vary markedly in shape, structure, and size [1,5]. Their variation in dehiscence is a focal point for research because it fundamentally changes fruit structure, subsequently affecting dispersal and diversification [6]. A prerequisite for exploring how differences in fruit morphology are achieved across the Brassicaceae is familiarity with both the fruit structure and underlying genetic pathways of an important member of the family: *Arabidopsis thaliana* [7,8]. As this species is a premier model, it provides an important basis of comparison to species with variable morphology. Arabidopsis fruits, hereafter referred to as typical siliques, are composed of five basic elements: valve, replum, seeds, septum, and valve margins. The valve, synonymous with ovary wall in Arabidopsis, is the outermost tissue of the fruit that protects the developing seeds and is separated from the replum at maturity to release seeds. The replum is the persistent placental tissue to which the seeds are attached. The septum, which connects to the replum, divides the fruit into two locules or chambers. The valve and replum are separated by the valve margin, which consists of a lignification and separation layer. Thus, proper fruit formation relies on the establishment of medial (replum) and lateral (valves and valve margin) components [9]. As the fruit dries, tension is created via the lignified layer, which facilitates the separation of the valves from the replum at the separation layer [10]. This general morphology is stable across most dehiscent members of Brassicaceae [1].

**Fig 1:**
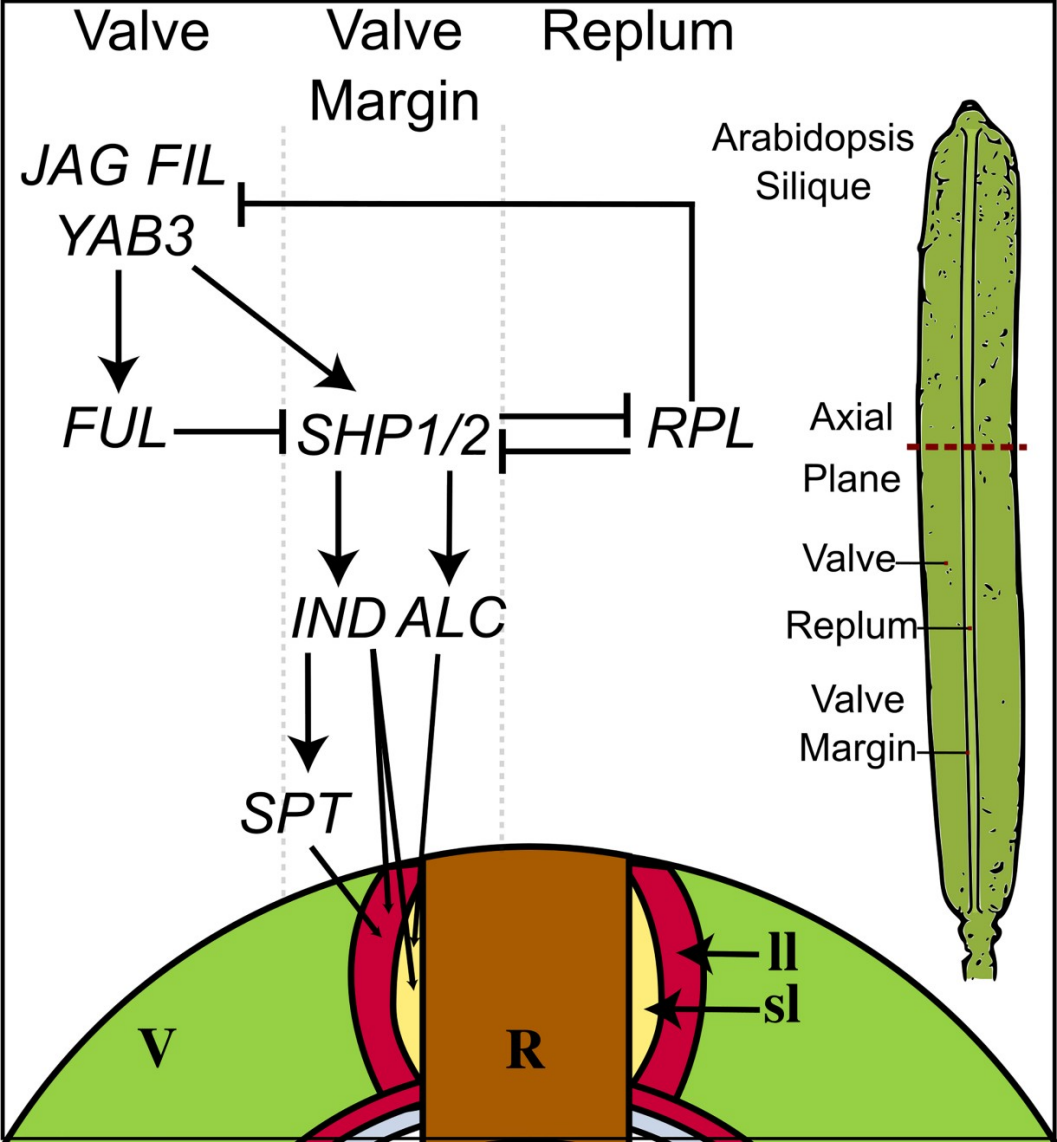
Diagram of simplified valve margin pathway for fruit dehiscence in *Arabidopsis thaliana;* valve margin. R, replum. Sl, separation layer. ll, lignification layer. Valve margin = sl + ll. Modified from data available in [7–8,11] and Fig 2 [34].

The causal factors for dehiscence have been well characterized in Arabidopsis [11–14], with proper formation and positioning of the valve margin being a key to this process. The valve margin pathway is essential for spatial regulation and development of valve, replum, and valve margin tissues [8,15–20]. Briefly, *FRUITFULL* (*FUL)* and *REPLUMLESS* (*RPL*), as well as other upstream regulators, restrict the expression of the valve margin genes to two cell layers between the valve and replum, respectively. The valve margin genes, *SHATTERPROOF 1/2* (*SHP1/2*), *INDEHISCENT* (*IND*), *SPATULA* (*SPT*), and *ALCATRAZ* (*ALC*), are responsible for the formation of the valve margin, specifically of the separation and lignification layers that control dehiscence (Fig 1). Upstream regulators of *FUL* and *RPL*, e.g., *APETALA2* (*AP2*), *FILAMENTOUS FLOWER* (*FIL*), *YABBY3* (*YAB3*), and *JAGGED* (*JAG*) are also key to precise positioning of the valve margin because they tightly regulate downstream processes. In sum, replum and valve genes function in an antagonistic manner to ensure proper formation of these regions of the fruit [9].

A common modification in fruit morphology across Brassicaceae is indehiscence, which has been observed in at least 20 lineages, implying multiple origins of this trait [21]. There are many genetic modifications that result in an indehiscent Arabidopsis fruit, but less is known about basis of indehiscence observed in other species. For example, a mutation in the following genes results in indehiscent fruits in Arabidopsis: *SHP1/2*, *SPT*, *ALC* and *IND* [22–25]. Overexpression of *FUL* or *NO TRANSMITTING TRACT* (*NTT*) also results in indehiscent fruits [26,27]. In studies of other species, fewer genes have been implicated in the indehiscent phenotype. One study demonstrated a deviation in expression of eight key genes between pod shatter sensitive species and shatter resistant species of *Brassica* and *Sinapis* [2]. In *Lepidium*, there has been an evolutionary shift from dehiscence to indehiscence, e.g., valve margin genes that are conserved between the dehiscent *L*. *campestre* and Arabidopsis have been lost in the indehiscent *L*. *apellianum* [28,29]. Upregulation in upstream regulator *AP2* has been suggested as a factor in this origin of indehiscence [29].

**Fig 2:**
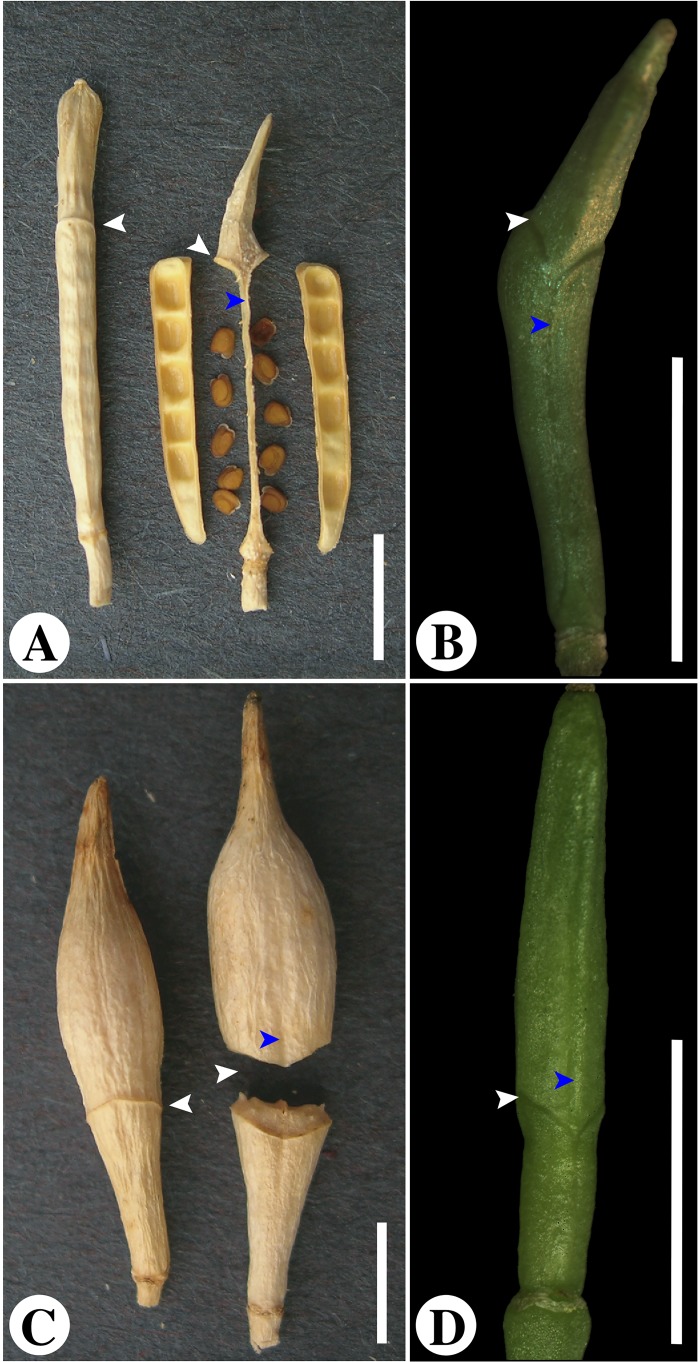
Mature and young heteroarthrocarpic fruits. (A), Mature *Erucaria erucarioides* fruit in lateral view before dehiscence (left), and medial view after dehiscence (right). (B), Young *E*. *erucarioides* fruit representing size sampled for transcriptomics in medial view. C, *Cakile lanceolata* fruit in lateral view before joint abscission (left), and medial view after joint abscission (right). (D), Young *C*. *lanceolata* fruit representing size sampled for transcriptomics in medial view; Modified from Fig 1 [34]. White arrows indicate joint region; blue arrows indicate replum. Scale bars = 5mm.

A notable morphological adaptation is the evolution of a complex fruit type known as heteroarthrocarpy, which is only found in some members of the tribe Brassiceae [21,30,31]. This modified silique is defined by the presence of a variably abscising central joint, an indehiscent distal region, and a variably dehiscent proximal region (Fig 2). As such, this novel morphology offers an opportunity to investigate fruit variation beyond shifts from dehiscent to indehiscent. Ancestral state reconstructions reveal that typical siliques are ancestral in the tribe with multiple origins to heteroarthrocarpy. However, reconstructions vary in the precise number of times this trait has evolved. In contrast, evolutionary patterns of dehiscence and joint articulation are less clear with closely related taxa exhibiting variation in these features [32]. Anatomically, heteroarthrocarpic fruits appear most like Arabidopsis siliques in their proximal regions, varying by a lack of a valve margin cell layer in indehiscent variants [32–34]. There are three described variations of heteroarthrocarpy: a non-abscising joint with a dehiscent proximal region, an abscising joint with an indehiscent proximal region, and an abscising joint with a dehiscent proximal region [34]. These subtypes have evolved multiple times, perhaps as a bet hedging strategy in response to selective pressure from hostile desert environments [6,32]. The evolution of the joint and subsequent heteroarthrocarpic subtypes may be developmental enablers that have facilitated changes in fruit morphology across the tribe, which would explain heteroarthrocarpy’s evolutionary lability [34]. Regardless of lability, all types are linked by the mechanism in which seeds from the same fruit are released by different means. In other words, the joint is the novel and unifying feature of heteroarthrocarpy [34].

A comparison of expression patterns between heteroarthrocarpic subtypes is potentially informative for formulating hypotheses about its evolutionary origins. *Erucaria erucarioides* and *Cakile lanceolata*, hereafter referred to as *Erucaria* and *Cakile*, are two well-studied representatives for heteroarthrocarpy because of their close relation and divergent subtypes [6,7,32,34]. In all heteroarthrocarpic subtypes the distal region remains indehiscent. *Erucaria* represents the subtype where the proximal region dehisces at maturity, releasing seeds, and the distal region remains attached to the mother plant via the persistent replum (Fig 2). *Cakile* exhibits the subtype where the proximal region also remains indehiscent with joint abscission such that all seeds are dispersed as protected propagules (Fig 2). In previous studies it was hypothesized that the formation of the joint is the result of repositioning of the valve margin, such that the valve is only present in the proximal region of the fruit, unlike in Arabidopsis where it is found in the entire ovary [34]. In other words, the joint is the distal portion of the valve margin. This hypothesis was partially supported by comparative gene expression data of some, but not all, genes in the valve margin pathway using a candidate gene approach [7]. However, that study did not definitively determine how the pathway has been repositioned because it did not investigate upstream genes. Candidate gene approaches will, by design, overlook non-targeted genes, and a lack of in situ hybridization does not necessarily indicate a lack of expression. Further, the basis of the joint remains unknown. No study to date has investigated transcriptional variation of heteroarthrocarpic fruits sectioned transversely into distal, joint and proximal regions. This approach is complementary to prior research because it quantifies expression of all transcripts in discrete regions of a whole system. Expression profiles from these regions will elucidate broad patterns and potentially identify key players involved in the formation of heteroarthrocarpy. They will clarify unique and shared gene expression patterns between and within *Erucaria* and *Cakile*, and will set the groundwork for future research regarding the evolution of the joint. Herein, the objective is to uncover transcript patterns, unique or shared, between and within, two variant heteroarthrocarpic species. We expect gene expression to be consistent with anatomical features within fruits, and that expression of fruit patterning transcripts will be consistent with repositioning of the valve margin in heteroarthrocarpy.

## Materials and methods

### Plant material

Seeds from *Erucaria erucarioides* (Coss. and Durieu) Müll. Berol and *Cakile lanceolata* (Willd.) O. E. Schulz were obtained from the late César Gómez-Campo’s and KEW royal botanical garden’s seed collections, respectively. Vouchers for *Cakile* and *Erucaria* have been deposited in the Vascular Plant Herbarium at the University of Alberta, and the Harvard University Herbaria, respectively. Seeds were germinated in 1% agar and transferred to clay pots containing a 2:1 soil (Sungro sunshine mix #4, Agawam, MA, USA) to perlite mixture. Plants were grown under a 16/8-hour light/dark schedule at 24°C with scheduled watering in the University of Alberta, Department of Biological Sciences, growth chambers.

Distal, joint, and proximal regions from 10mm fruits (~10 days post fertilization) were collected and flash frozen in liquid nitrogen prior to storage at -80°C. Distal and proximal regions were classified as all tissue ~1mm above or below the joint, and the joint is remaining tissue between distal and proximal regions (Fig 2). The 10mm fruit size is roughly equivalent to Arabidopsis stage 17A fruits [35], which go through elongation and cell expansion before maturity. This size was chosen to capture late stage valve margin gene expression because the valve margin is easily distinguished at this stage, and an increase in lignification is observed in key layers, e.g., en*b*. [36].

### RNA isolation and cDNA library preparation

RNA was extracted from frozen tissue using manual grinding and a Qiagen RNeasy micro kit (Hilden, Germany) with the following amendments to protocol: RNA was incubated in nuclease free water for five minutes prior to elution, and this eluate was spun through the same extraction column to maximize RNA yield. RNA concentration was verified using a Nanodrop ND-1000 spectrophotometer (Software version 3.1.2), and quality was confirmed using the Agilent 2100 bioanalyzer (Software version B.02.09.SI720). All cDNA samples were set at the same concentration of the most dilute RNA extraction. Samples were processed using the Illumina TruSeq stranded mRNA LT sample prep kit RS-122-2101 (California, U.S.), and the procedure was followed as described in the low sample protocol. The mRNA from each sample was isolated and purified using AMPure XP magnetic beads (Agencourt; Beverly, Massachusetts) before primary and secondary strand cDNA synthesis. Unique Illumina adapters were ligated, and each sample was PCR amplified before validation. PCR was run for 15 cycles of: 98°C for 10 seconds, 60°C for 30 seconds and 72°C for 30 seconds followed by 5 minutes at 72°C and a final hold at 4°C. Samples were normalized, pooled, and sequenced by the center for applied genetics (TCAG) facilities of the Toronto Sick Kids hospital, Ontario, Canada.

### De novo transcript assembly, differential expression, and annotation

Raw reads were trimmed and quality checked using Trim Galore! (Version 0.4.1) [36] and FastQC (Version 0.11.3) [37] then assembled using Trinity (Version 2.2.0) [38]. The raw reads are available at the Sequence Read Archive (SRA) database under the BioProject ID PRJNA545186. The Transcriptome Shotgun Assembly projects have been deposited at DDBJ/EMBL/GenBank under the accessions GHNY00000000 and GHOR00000000 for Erucaria and Cakile, respectively. The versions described in this paper are the first versions, GHNY01000000 and GHOR01000000. Corset (Version 1.0.6) [39] was used to estimate contig abundance by grouping contigs into representative gene clusters as the first step of the differential expression analysis. Contigs are defined as continuous overlapping paired-end reads. Next, edgeR (Version 3.6.2) [40,41] was used to perform pairwise differential expression analysis of Trinity gene, Trinity contig, and Corset clusters between proximal, joint, and distal regions of fruits from the same species. Genes, contigs, and clusters were classified as significantly differentially expressed if log2(fold-change) was greater than 2 and the False Discovery Rate (FDR)-corrected p-value (α) was less than 0.05. The analyze_diff_expr.pl script, provided with Trinity, was used to generate z-score heatmaps of all significantly differentially expressed contig clustered transcripts (α < 0.05). A z-score is used to indicate how many standard deviations a value is above the mean. The transcriptomes were annotated using the Basic Local Alignment Search Tool (BLAST) [42] algorithm on a local copy of both the National Center for Biotechnology Information (NCBI) non-redundant protein (nr) database and The Arabidopsis Information Resource (TAIR) 10 database [43]. BLASTx (E-value<10^−10^) was used to identify highly similar sequences, and transcripts with the highest bit-score from the TAIR database were used as representative transcripts for heatmap generation. Whole transcriptome and fruit patterning heatmaps were generated using ggplot2 [44] and ggplot in R, respectively (Version 3.4.2) [45]. These global gene expression patterns were compared to previously published in situ hybridization and semi-quantitative PCR of select fruit genes [7]. Assembly completeness was determined using Benchmarking Universal Single Copy Orthologs (BUSCO) (Version 2.0) [46] (S1 Table).

### Orthologous clustering

Orthofinder (Version 1.1.8) [47] was used to group orthologous transcripts from unfiltered *Erucaria* and *Cakile* transcriptomes. These groupings (orthogroups) with transcripts from both species as well as top BLAST matches for fruit patterning genes of interest were used to generate heatmaps. For Venn diagram generation, high-throughput sequencing (HTS) [48] filtered transcripts, sorted by regions, were translated to longest open reading frame (ORF) protein fasta files using TransDecoder (Version 5.0.0) [49]. These files were uploaded for comparison using the Orthovenn webserver [50]. HTS filtering was used to reduce file size due to the web server upload limit, and to reduce the number of insubstantial transcripts.

### Gene ontology

Transcriptome fasta files from *Erucaria* and *Cakile* were imported to BLAST2GO (Version 2.8) [51]. Annotation files were exported and filtered, using a merged profile of all three biological replicates, to generate gene ontology (GO) terms for each region and species. These GO terms were used to produce graphs containing transcriptome hits for chosen terms. Terms were chosen based on searches for lignin, abscission, dehiscence, specific hormone keywords, and top hits. For comparison between transcriptomes, the log2 of selected GO term counts were divided over the log2 of all GO term counts (log2(n)/log2(N)).

## Results

### De novo assembly of *Erucaria* and *Cakile* transcriptome data

RNA-seq libraries were constructed from 9 total replicates of triplicate distal, proximal, and joint regions. RNA samples from segmented fruits of two distinct plants were combined before sequencing to achieve optimal yield for library preparation. Sequencing from both libraries averaged 27.41 and 29.41 million paired-reads for *Erucaria* and *Cakile*, respectively. After quality trimming read counts were reduced to 27.36 million and 28.36 million high quality reads, respectively. Inter-quartile ranges per base were minimally 33 for *Erucaria* for the first 5 base pairs, and minimally 32 in the 90^th^ percentile; *Cakile’s* inter-quartile ranges were minimally 33 for the first 5 base pairs, and minimally 29 in the 90^th^ percentile.

The transcriptome from *Erucaria* had an average contig length of 942.83, and *Cakile’s* had an average length of 877.15. The total transcript count for *Erucaria* and Cakile was 227,530 and 314,194 reads, respectively (Table 1). Corset cluster counts averaged 365,257 (*Erucaria*) and 436,177 (*Cakile*). Notably, the first replicate for *Cakile* had a read count of 269,732, which is minimally 130,000 fewer than replicate 2 and 3. This inconsistency may have caused some issues in downstream analyses, but overall, both transcriptomes were of adequate quality and read-depth. This is supported by a BUSCO analysis, as Erucaria and Cakile’s assemblies had overall completeness of 96.4% and 94.8%, respectively (S1 Table).

**Table 1:** Statistics for de novo Trinity assembly of *Erucaria erucarioides* and *Cakile lanceolata* pairwise reads for all isoforms. Numbers in parentheses refer to longest isoform only.

	*Erucaria*	*Cakile*
**N50**	1544 (1017)	1464 (835)
**Median Contig Length**	578 (374)	517 (330)
**Average Contig length**	942.83 (656.94)	877.15 (577.55)
**Total Assembled bases**	214,521,562 (92,098,767)	275,595,508 (108,815,069)
**Total Trinity Genes**	140194	184945
**Total Trinity Transcripts**	227530	314194
**GC%**	41.89	42.05

### Annotation of assembled transcripts

Both transcriptomes were compared to the nr and TAIR peptide database using a BLASTx algorithm, and all downstream analyses used the TAIR10 annotation for facilitated comparison to Arabidopsis. A total of 254,592 (*Cakile*) and 213,757 (*Erucaria*) transcripts with e-values≤ 10^−5^ were matched to the TAIR10 database with multiple transcripts matches per gene. The GO analysis averaged 8,644 and 8,941 terms for *Erucaria* and *Cakile*, respectively. The top 15 GO terms consisted of 11 cellular component, three molecular function, and one biological process. Nucleus, plasma membrane, and protein binding were the top three terms, all of which are biological processes (S1 Fig).

**Fig 3:**
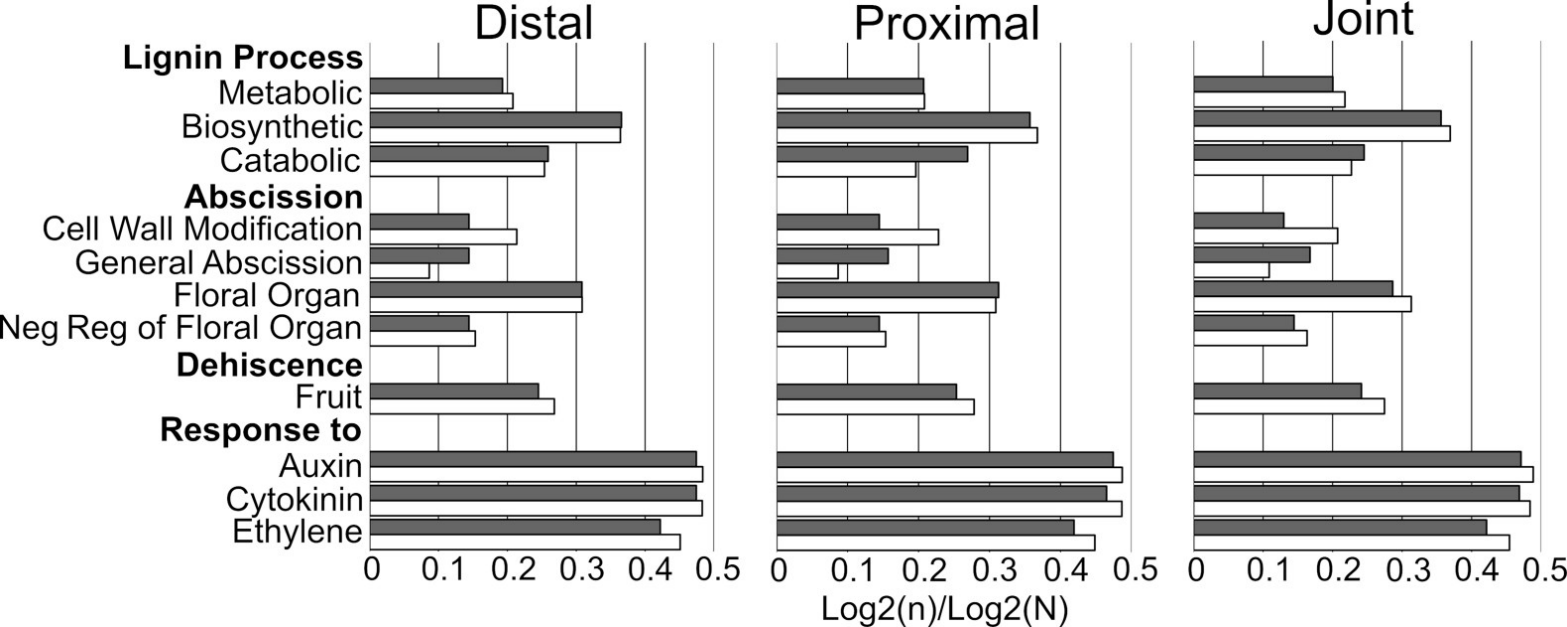
Graph of select Gene Ontology (GO) terms for *Erucaria erucarioides* (grey) and *Cakile lanceolata* (white). GO counts based on merged profile of three biological replicates per region. Sample (n) and total (N) raw counts log2 transformed for interspecies comparison. GO terms chosen based on search terms: lignin, abscission, dehiscence, and response to hormone.

The majority of selected orthogroups were similar between and within species (lignin, abscission, and dehiscence processes, and hormone response) (Fig 3). Exceptions include: cell wall modification related to abscission, general abscission, and catabolic lignification. *Cakile* has a greater ratio of cell wall modification processes and a lower ratio of general abscission processes relative to *Erucaria*. *Erucaria* has a higher ratio of catabolic lignification processes in the joint region despite having similar ratios relative to *Cakile* in the distal and proximal regions (Fig 3). Overall, the GO analysis results are consistent between and within species.

**Fig 4:**
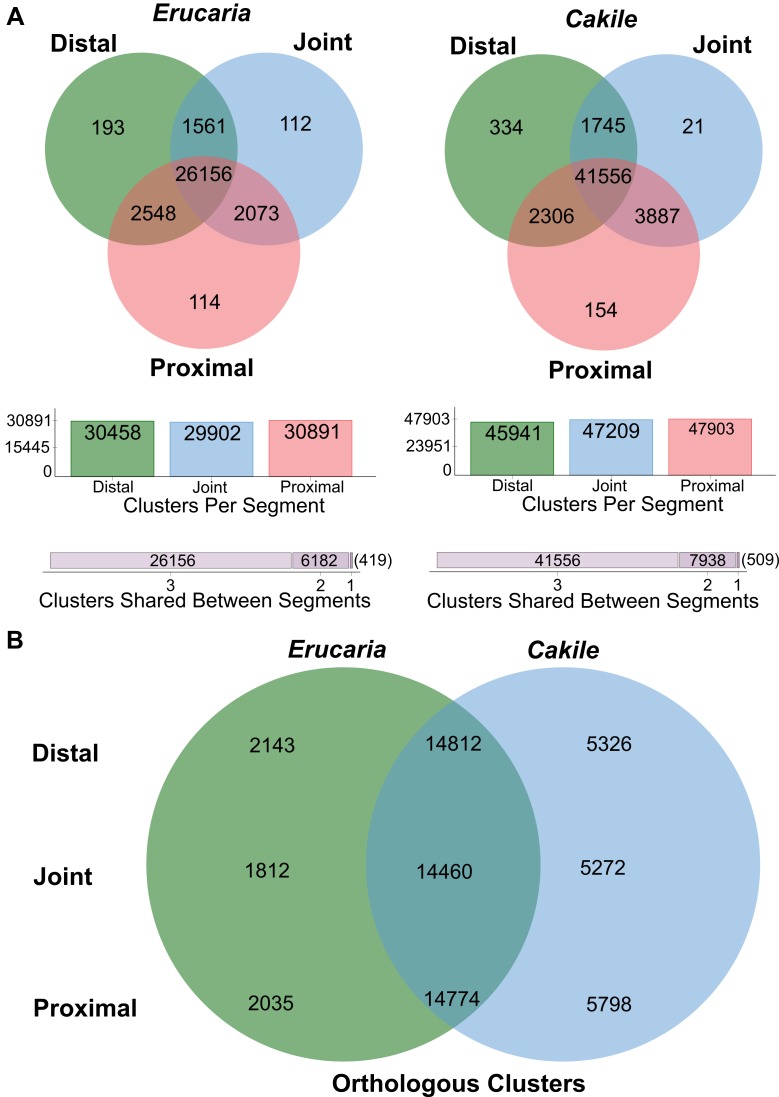
Venn diagrams of three-way and pairwise High Throughput Sequencing (HTS) filtered transcripts for *Erucaria erucarioides* and *Cakile lanceolata* transcriptomes. (A), Three-way Venn diagrams of *Erucaria* and *Cakile* orthologous clusters for distal, joint, and proximal regions. (B), Pairwise Venn diagrams of *Erucaria* and *Cakile* orthologue-clustered transcripts (*Erucaria* region vs *Cakile* region).

Additional results from OrthoVenn showed minimal difference in orthologous clustering within species, but some differences between species (Fig 4). There are a greater number of shared clusters between the proximal and distal regions in *Erucaria* (2548) than *Cakile* (2306) despite *Cakile* having substantially more overall clusters than *Erucaria* (50,003 vs 32,757). Additionally, there are fewer clusters unique to the joint for *Cakile* (21) than *Erucaria* (112). In sum, there are fewer orthologous clusters in common within regions of *Cakile* fruits than within regions of *Erucaria* fruits.

### Identification of differentially expressed transcripts in 10mm fruit

**Fig 5:**
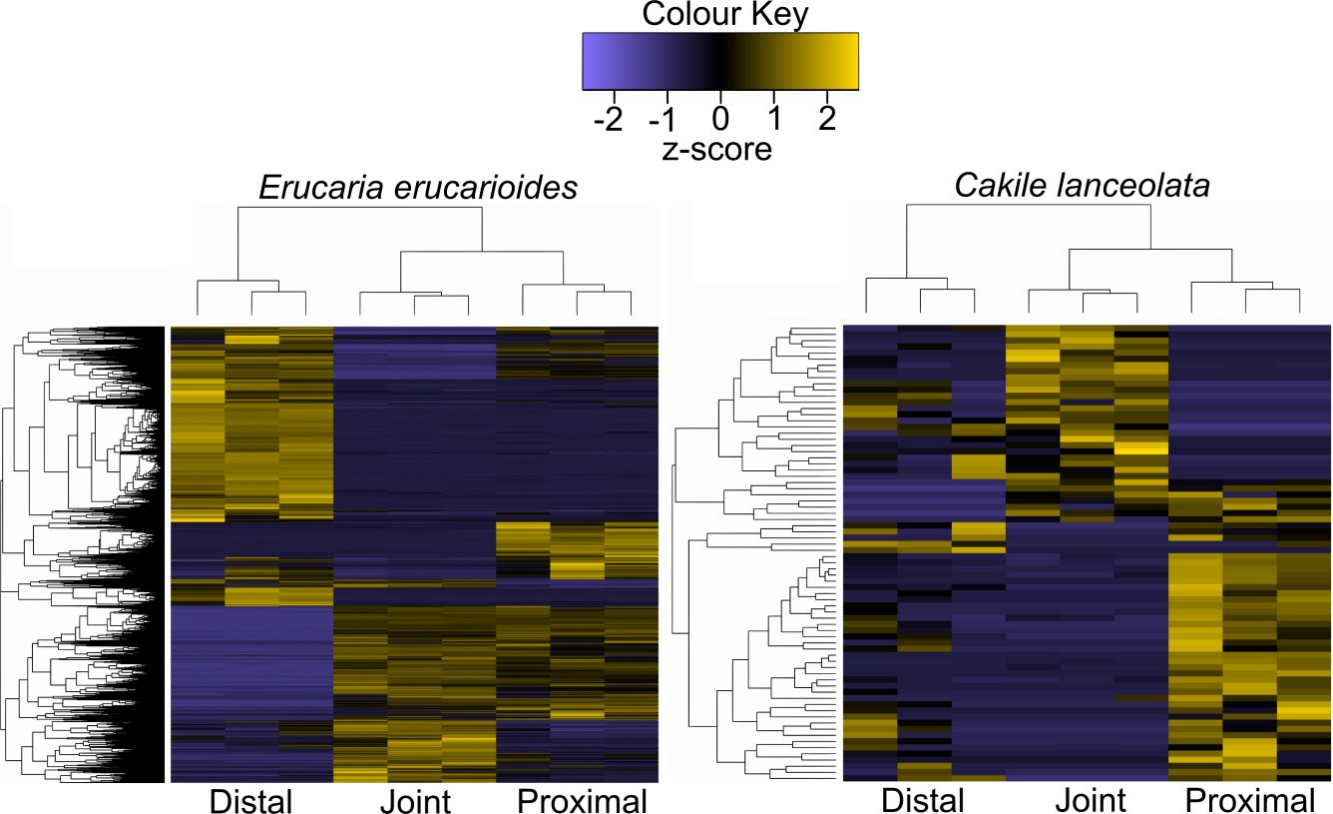
Heatmap of all significant edgeR contig clustered transcripts in the *Erucaria erucarioides* (n = 15,345) and *Cakile lanceolata* (n = 74) transcriptomes, expressed as z-scores (FDR-corrected α = 0.01).

For whole transcriptome comparison, two heatmaps of significant pairwise differentially expressed transcripts (α = 0.01) were generated (Fig 5). Contig clustering was chosen for this analysis because it is a more conservative estimation of significant differential expression at the transcript level, i.e., there are a greater number of transcripts being compared with more stringent FDR correction relative to corset clustering. Values were then converted to z-score to facilitate interspecies comparison, and for visual clarity. Dendrograms highlight the differences in number of differentially expressed transcripts between both species, and show that all replicates clustered together, respectively. The joint and proximal regions of *Erucaria* are most alike in expression and are both dissimilar to the distal region (Fig 5). All three regions in *Cakile* have different expression patterns, and the distal region has a relatively large inter-replicate variance (Fig 5). There are 15,345 (*Erucaria*) and 74 (*Cakile*) significantly differentially expressed (SDE) transcripts in each transcriptome. There were no SDE *Cakile* transcripts with FDR-adjusted p-values < 0.01. The low number of SDE genes between *Cakile* regions indicates a lack of regional distinction in terms of transcript expression. These data demonstrate a large difference in significant differential expression between the distal region relative to the joint and proximal region in *Erucaria*, and little significant variation between all three *Cakile* regions.

**Fig 6:**
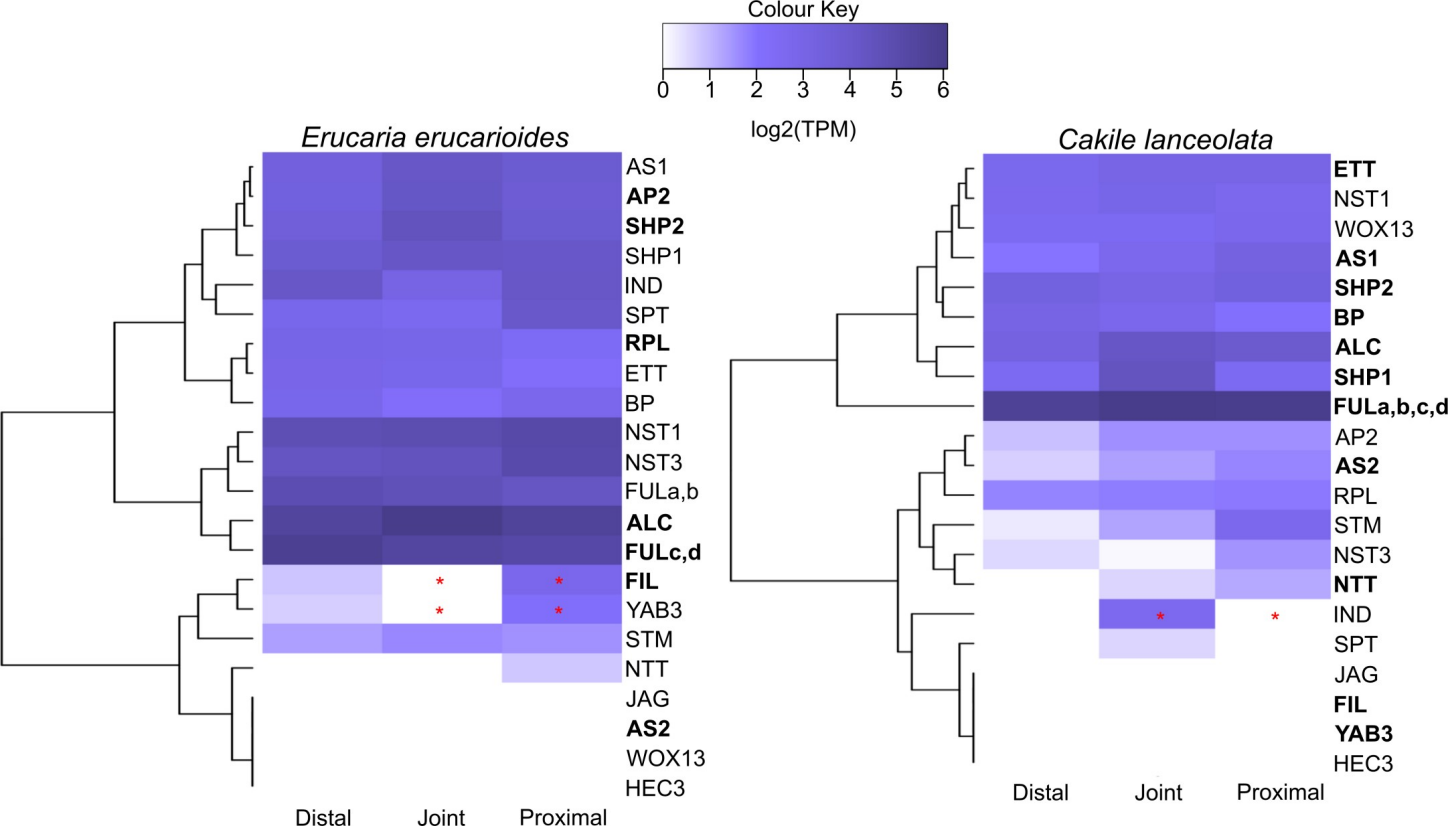
Heatmap of edgeR contig clustered transcripts from *Erucaria erucarioides* and *Cakile lanceolata* expressed in log2 (TPM) with TMM normalization. Representative transcripts are those with the highest bitscore hit against the TAIR database. Bolding indicates shared orthogroup with other transcriptome. FULa,b,c,d are copies of FUL that are present in some species across the Brassicaceae [71]. TPM, Transcripts Per Million; TMM, Trimmed Mean of M-values. Asterisks indicate significant differential expression between proximal and joint region. (FDR-corrected α = 0.01).

We compared expression profiles of 21 genes important for valve margin formation and positioning in Arabidopsis [2,11,16,52–63] (Fig 6). Contig clustered transcripts were also chosen for this analysis based on matches against the TAIR database. Row dendrograms highlight the different clustering of expression patterns of VM genes between both species, e.g., *ETT*, *RPL* and *BP* cluster together in *Erucaria* but not in *Cakile* (Fig 6). Most fruit patterning genes for both species have no significant differences in expression across all regions, except for *FIL* and *YAB3* which were significantly upregulated in the distal region relative to the joint in *Erucaria*, and *IND* which was significantly upregulated in the joint relative to both the distal and proximal regions in *Cakile*. Upstream regulators *FIL* and *YAB3* are not expressed in late stage *Cakile* fruits, despite global expression in *Erucaria* fruits. Downstream regulator *IND* is expressed in the whole fruit in *Erucaria*, but only in the joint region of *Cakile* (Fig 6).

## Discussion

### Gene ontology of heteroarthrocarpic fruits

Overall, GO terms within fruits and between species are similar (Fig 3 and S1 Fig), as expected, because all sections and replicates are from developing fruit with shared components (e.g., ovary wall, septum). Additionally, GO analyses of top terms do not usually vary between closely related species [64,65]. However, despite similarities in gene ontology, the origin of heteroarthrocarpy may still be explained by deviation in expression patterns of one or more of the valve margin pathway genes [18,22–26]. Similarities in gene ontology do not imply similarity between all expressed transcripts, so variation of just a few transcripts may be the driving factor behind heteroarthrocarpy.

### Global transcript expression of heteroarthrocarpic fruits are consistent with anatomy

Transcript expression patterns are consistent with anatomical variances within and between fruits. The distal region of *Erucaria* has opposing transcript expression relative to both its joint and proximal regions (Fig 5), i.e., when transcripts are upregulated distally in *Erucaria* they are downregulated proximally. This pattern is consistent with heteroarthrocarpic fruit anatomy, as distal regions contain no valve or valve margin, and proximal regions have both [34]. In contrast, all regions of *Cakile* have variable transcript expression, with the clearest distinction between the proximal and joint regions, i.e., when genes are upregulated proximally they will be downregulated in the joint (Fig 5). As with *Erucaria*, expression profiles in *Cakile* vary in a manner consistent with anatomy. Superficially, one might expect the *Cakile* silique to have similar expression between all regions because the entire fruit is indehiscent, which is consistent with the pattern of significantly fewer DE genes between regions of *Cakile* (74) than *Erucaria* (15,345) (Fig 5). However, anatomically, the distal region of *Cakile* is more similar to the distal region of *Erucaria* than to its own proximal region [34], and its abscising joint is anatomically reminiscent to a valve margin [34]. Thus, we would expect regions of the fruit to exhibit different expression patterns, which is supported by our data (Fig 5). Abscission zones are also found between septum and seeds, and they too share similar anatomy and expression to typical silique valve margins [66]. Heteroarthrocarpic distal regions are unlike indehiscent non-heteroarthrocarpic siliques such as *L*. *appelianum*, because heteroarthrocarpic distal regions have no remnant valve margin in contrast to indehiscence observed in *Lepidium* and the proximal region of *Cakile* [29,34]. Thus, we expect different expression patterns within heteroarthrocarpic fruits, as well as between heteroarthrocarpic and non-heteroarthrocarpic fruits. In summary, there is a clear difference between distal and proximal expression profiles for both *Erucaria* and *Cakile*, which is consistent with a repositioning of the valve margin, i.e., the distal region is quite distinct from the proximal region due to the lack of valve margin, or its remnant, in the distal region. This consistency is further explored by analysis of fruit patterning transcript expression involved in valve margin formation.

### Fruit patterning genes

Despite the substantial differences in anatomy, most valve margin genes reveal similar expression patterns across fruits in both *Erucaria* and *Cakile* (Fig 6). Although overall these expression patterns are consistent with a previous investigation of some, but not all, members of the VM pathway [10], they vary with regards to expression of one gene in *Erucaria*. *E*e*FUL1*, one of two *FUL* homologs found in *Erucaria*, was previously shown to only be expressed in the proximal region in earlier stages of carpel development [7], but all *FUL* transcripts are expressed across all regions in this study of later stage development (Fig 6). This discrepancy may be due to dynamic gene expression at different stages or because our methodology cannot distinguish within region differences (e.g., genes expressed in valve but not replum), so differences within regions cannot be distinguished. In contrast to *EeFUL1*, our data are consistent with a previous publication which demonstrated that other fruit patterning genes have broader expression domains than observed in Arabidopsis [7]. *EeALC* and *EeIND* and *ClALC* were expressed in the septum of *Erucaria* and *Cakile*, respectively, which is found throughout all regions sampled in this study.

It is a compelling finding that upstream regulators *FIL*/*YAB3* and *JAG* have variable expression across *Erucaria* (Fig 6). These three genes positively regulate expression of *FUL* and valve margin genes in Arabidopsis such that their cooperative function has been designated together as *JAG/FIL* activity [19]. Our data suggest a decoupling of this cooperation in heteroarthrocarpic fruits because these three genes do not exhibit the same expression patterns across *Erucaria* fruits (Fig 6). That is, no expression of *JAG* was detected in any region of either species at this stage. *FIL* and *YAB3* showed different expression patterns across fruits of *Erucaria*, but neither were detected in *Cakile*. It is important to note that plants of double mutants’ *fil*/*yab3* in Arabidopsis have fruits that are remarkably reminiscent of heteroarthrocarpy, e.g., they lack valve margin in the distal region of fruit while maintaining ovary wall identity [8]. In contrast to heteroarthrocarpy, these mutants have ectopic valve margin in the proximal region of their fruits [8]. As these genes exhibit different patterns across *Cakile* and *Erucaria* and are expressed in both proximal and distal regions of *Erucaria*, heteroarthrocarpy cannot be explained by a simple lack of expression of these key regulators. Further, *FIL/YAB* are absent in the joint region of *Erucaria* (Fig 6), which is confounding since the joint contains small portions of both proximal and distal regions, an unavoidable consequence of segmentation during tissue collection. Nonetheless, deviation in expression patterns of these upstream regulators between Arabidopsis and heteroarthrocarpic fruits implicates variation in their expression profiles in the origin of heteroarthrocarpy.

When exploring heteroarthrocarpy, we need to consider fruit patterning beyond the basal-apical differences that distinguish distal, joint, and proximal regions. That is, the medial (replum) patterning (Fig 1) is maintained in heteroarthrocarpic fruits whereas the lateral is not. This pattern is due to differences in dehiscence between proximal and distal segments: undifferentiated ovary wall is present in the distal region whereas valve or remnant valve is present in proximal region. In other words, replum tissue is present in distal, joint, and proximal regions of heteroarthrocarpic fruits regardless of whether the ovary wall has differentiated into valve and it is appropriately sized [36]. *FIL*/*YAB3* and *JAG* function antagonistically with replum promoting gene, *WUSCHEL RELATED HOMEOBOX 13* (*WOX13*), which positively regulates *RPL* in turn. This interaction is necessary for proper medial-lateral formation of Arabidopsis fruits. Further, *ASYMMETRIC LEAVES1* (*AS1*) and *AS2* collaborate with *JAG*/*FIL* function as promoters of lateral factors [9]. The loss of both *AS1*/2 and *JAG*/*FIL* in Arabidopsis results in dramatic medial-lateral differences and substantially enlarged replum, which is interestingly more pronounced in the basal portion of the fruit [9,67]. As *AS1*/2 and *AS1* are expressed throughout *Cakile* and *Erucaria* regions, respectively, this pattern suggests that *AS1* alone is sufficient for proper replum (aka medial-lateral) formation in heteroarthrocarpic fruits. In other words, the collaboration between *JAG*/*FIL* function and *AS1*/2 is not maintained in heteroarthrocarpic fruits. Further, *JAG/FIL* activity is non-detectable in the entire fruit, at least in *Cakile* at later stages of development. Thus, it appears that some redundancy in lateral-medial patterning of Arabidopsis fruits has been lost in heteroarthrocarpic fruits, as supported by the different clustering of VM genes for each species (Fig 6), while simultaneously gaining apical-basal differences, e.g., dehiscence and indehiscence in the proximal and distal regions of *Erucaria*.

### Valve margin pathway recruitment for abscission of the Cakile joint

The fruit of *Cakile* is distinct in that the joint abscises (disarticulates) at maturity. The joint, which represents the distal portion of the valve margin, thus represents a novel abscission zone in *Cakile*, completely separating the distal portion of the fruit. This is an unusual feature of certain heteroarthrocarpic subtypes, as there is no equivalent abscission zone in Arabidopsis. Our data strongly implicate the recruitment of downstream valve margin genes as responsible for joint abscission, although how that zone is positioned remains elusive. *IND* is significantly upregulated in joint region (Fig 6) and is primarily responsible for formation of separation and lignification layers in typical siliques [22,24], a juxtaposition of cell types also observed in the abscising joint region. Its presence in the joint may be due to a co-option of downstream valve margin pathway genes to facilitate formation of the joint abscission zone. Similar co-option is observed in seed abscission zones, although these zones typically involve *SEEDSTICK* (*STK*) in lieu of *SHP*, and the functionally similar transcription factor *HEC3* in lieu of *IND* [66]. *SHP1/2* and *ALC* expression are both consistent with this co-option, as they are expressed in all three regions (Fig 6). Additionally, *SPT* expression is consistent with expression of *IND*, as expected from its downstream role in valve margin formation (Fig 6) [11]. Further, both representative transcripts are among the 21 unique orthologous clusters in the joint of *Cakile* (Fig 4). This pattern is consistent with in situ hybridization data that showed *SHP2* expressed in septum and ovules of *Cakile*, and in ovules of *Erucaria* [7]. Thus, the likely function of *SHP1/2* and *ALC* in the joint region would be to promote expression of *IND* (*SHP1/2*), and the formation of the separation layer (*ALC*). What is unusual about joint abscission is that for the joint to separate, the distal and proximal regions of the replum must also separate. This expression pattern then implies that the mechanism used to physically separate valve from replum may also be in play for replum in the joint region. Taken together with anatomical studies, our data strongly suggests that there is a repurposing of the valve margin pathway in an otherwise indehiscent *Cakile* fruit, and that this pathway may be capable of initializing disarticulation in multiple tissue types.

## Conclusion

Transcriptomic expression from late stage *Erucaria* and *Cakile* fruits is consistent with some conservation and some deviation of the valve margin pathway, specifically in upstream regulation, e.g., *FIL/YAB3 and JAG*. Thus, different upstream regulators are implicated in the loss of dehiscence in Brassiceae relative to *Lepidium*, where *AP2* is likely responsible [29]. Loss of expression of *FIL/YAB3 and JAG* in Arabidopsis results in differing apical and basal phenotypes, which may help to explain the apical/basal differences in heteroarthrocarpic fruits [8]. Further, heteroarthrocarpic fruits likely recruit the same mechanism used in valve and seed abscission for joint abscission (Fig 6). Functional tests are necessary to confirm whether redeployment of *FIL*/*YAB3*, *IND*, and possibly *SPT* have key roles in the origin of heteroarthrocarpy as well as joint abscission.

There have been multiple whole genome duplications in the Brassicales, which has resulted in many polyploids within the Brassicaceae family [68–70]. We considered the possibility of transcriptional differences between gene copies in distal, joint, and proximal regions that were undetected because we were unable to determine copy number in our transcriptome. For example, there are four copies of *FUL* in the Brassiceae [71], but each potential *FUL* copy had multiple hits from the same transcripts in both transcriptomes, so there is no definitive answer about copy number and expression (Fig 6). That is, we could not confirm or refute subfunctionalization of some fruit patterning genes as having a role in the origin of heteroarthrocarpy. An analysis of multiple transcripts for every fruit patterning gene showed generally similar expression for each, but further analyses are needed to determine if neo/subfunctionalization plays a role in heteroarthrocarpy.

Understanding the nature of heteroarthrocarpy, and how it relates to fruit development in Arabidopsis, will facilitate future studies on seed shattering in important Brassicaceous crops, and pernicious heteroarthrocarpic weeds. Further, these studies inform on the origin of important variation in seed packaging and dispersal capabilities.

## Supporting information

S1 Fig Graph of top Gene Ontology (GO) terms for *Erucaria erucarioides* (blue) and *Cakile lanceolata* (white). Sample (n) and total (N) raw counts were log2 transformed for interspecies comparison.
(TIF)

S1 Table Benchmarking Universal Single Copy Ortholog (BUSCO) analysis of *Erucaria erucarioides* and *Cakile* transcriptomes. (DOCX)
