# Supplementary figures and images for: How to build a fruit: Transcriptomics of a novel fruit type in the Brassiceae
Source: PLoS One. 2019 Jul 18;14(7):e0209535. doi: 10.1371/journal.pone.0209535 (PMC6638736; doi:10.1371/journal.pone.0209535)

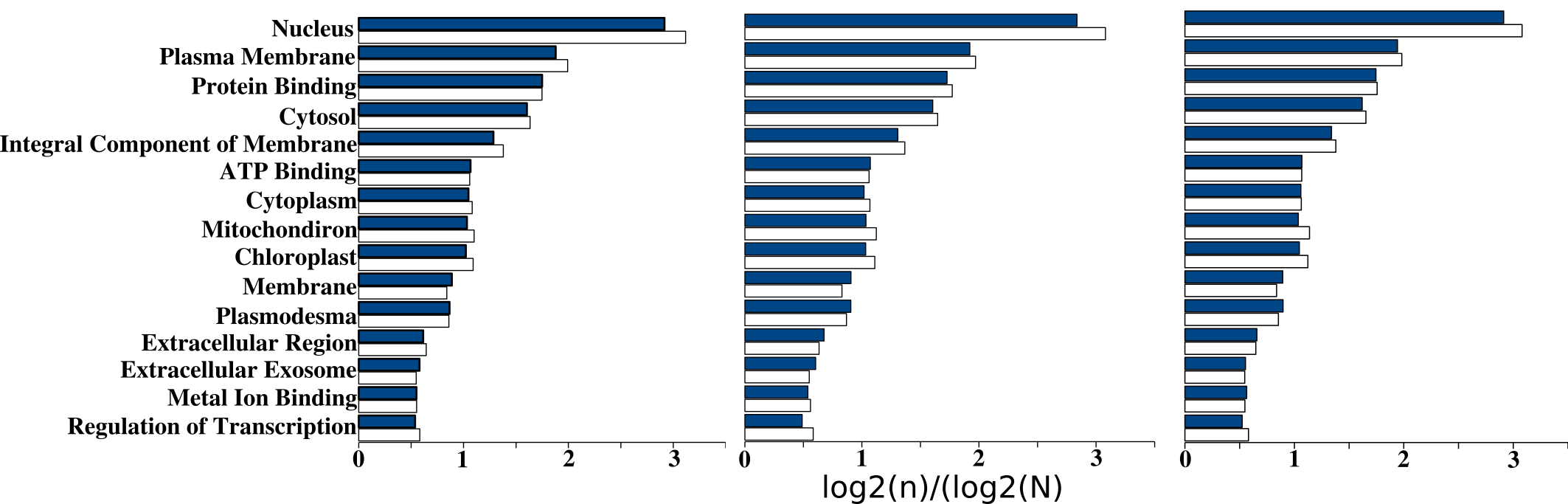

Supplement: S1 Fig — Graph of top Gene Ontology (GO) terms for Erucaria erucarioides (blue) and Cakile lanceolata (white). Sample (n) and total (N) raw counts were log2 transformed for interspecies comparison. (TIF) [file pone.0209535.s001.tif]
