# Supplementary material for: How to build a fruit: Transcriptomics of a novel fruit type in the Brassiceae
Source: PLoS One. 2019 Jul 18;14(7):e0209535. doi: 10.1371/journal.pone.0209535 (PMC6638736; doi:10.1371/journal.pone.0209535)
Supplement: S1 Table — (DOCX) [file pone.0209535.s002.docx]

|  | ***Erucaria*** | ***Cakile*** |
| --- | --- | --- |
| **Complete BUSCOs** | 1388 (96.4%) | 1366 (94.8%) |
| **Complete and single-copy (BUSCOs)** | 487 | 532 |
| **Complete and duplicated BUSCOs** | 901 | 834 |
| **Fragmented BUSCOs** | 33 (2.3%) | 43(3.0%) |
| **Missing BUSCOs** | 19 (1.3%) | 31(2.2%) |
| **Total BUSCO groups searched** | 1440 | 1440 |
